# A phase 2 randomized controlled trial using biologics to improve multi OIT outcomes (COMBINE): design, rationale, and methods

**DOI:** 10.3389/falgy.2025.1729111

**Published:** 2025-12-18

**Authors:** A. J. Long, S. Sindher, K. Martinez, J. H. Choi, M. Albarran, J. Schuetz, A. Parry, J. Tang, M. Garcia Llorett, S. S. Zedeck, A. Grissinger, E. Kiernan, S. Leonard, O. Raeber, C. Feight, B. Anderson, R. Sharma, D. Bogetic, A. R. Chin, M. Woch, J. Poyser, J. Laurienzo Panza, A. Togias, L. Wheatley, S. Boyd, S. J. Galli, K. C. Nadeau, R. S. Chinthrajah

**Affiliations:** 1 Department of Pathology, Sean N. Parker Center for Allergy and Asthma Research, Stanford University, Stanford, CA, United States; 2 Department of Pediatrics, Division of Immunology, Allergy, and Rheumatology, University of California, Los Angeles, CA, United States; 3 Division of Pediatric Allergy & Immunology, University of California, San Diego, CA, United States; 4 Rady Children’s Hospital, San Diego, CA, United States; 5 National Institute of Allergy and Infectious Diseases, National Institutes of Health, Bethesda, MD, United States; 6 Departments of Pathology and of Microbiology and Immunology, Stanford University, Stanford, CA, United States; 7 Department of Environmental Health, Harvard T.H. Chan School of Public Health, Boston, MA, United States

**Keywords:** biologic, COMBINE, dupilumab, food allergy, multi-allergen, omalizumab, oral immunotherapy

## Abstract

**Background:**

Food allergy remains a serious public health concern associated with significantly lowered quality of life and the risk of potentially life-threatening allergic reactions. While oral immunotherapy (OIT) has consistently demonstrated efficacy in the desensitization of multi-food allergic patients, many patients undergoing such treatment are burdened by dose-related side effects that can hinder their compliance and the overall efficacy of OIT. Recent efforts to improve upon OIT have begun to evaluate the concomitant use of biologics such as omalizumab and dupilumab with OIT for their ability to selectively inhibit pathways involved in the underlying pathology of food allergy.

**Methods:**

Herein, we detail the clinical trial design, rationale, and methods for a Phase 2 randomized, double-blind, placebo-controlled, multi-center study evaluating the safety and efficacy of omalizumab and/or dupilumab therapy in combination with participant-specific multi-food (mOIT) in patients aged 4–55 years, with multi-food allergy that includes peanut. In this two-arm superiority trial, participants will be randomized (5:5:1) to (1) omalizumab/placebo-dupilumab with mOIT (*n* = 50), (2) omalizumab/dupilumab with mOIT (*n* = 50), or (3) a mechanistic-only arm of placebo-omalizumab/dupilumab with mOIT (*n* = 10). Double-blind placebo-controlled food challenges (DBPCFCs) will be used to assess desensitization to ≥1,043 mg cumulative protein at Week 32, after which all treatment is to be discontinued. A follow-up assessment of sustained unresponsiveness via DBPCFCs will be conducted at Week 44.

**Conclusion:**

This trial tests the hypothesis that adding dupilumab to omalizumab-facilitated mOIT will increase the likelihood of inducing sustained unresponsiveness and decrease mOIT-related adverse events.

## Introduction

Food allergy (FA) remains a serious public health concern associated with significantly lowered quality of life and the risk of potentially life-threatening reactions. The prevalence of FA in the United States has increased substantially and ∼45% of patients are allergic to more than one food (1–4). There are currently 2 FDA-approved therapies for food allergy aside from life-long allergen avoidance: omalizumab and peanut oral immunotherapy (OIT).

Omalizumab is a recombinant humanized immunoglobulin G1 kappa monoclonal antibody that selectively binds to human IgE. Omalizumab inhibits the binding of IgE to the high-affinity IgE receptor, Fc*ε*RIa, on the surface of mast cells and basophils and reduces levels of free IgE in the blood (5). Treatment with omalizumab leads to protection from accidental ingestion of 1,000 mg of multiple foods in up to two-thirds of patients (6). However, given that omalizumab is neither curative nor disease modifying and is indicated for use alongside allergen avoidance, patients may need to continue taking the omalizumab indefinitely unless the food allergy is outgrown.

In contrast to omalizumab, OIT may be disease modifying and can induce long-term immune changes in a subset of patients. Landmark studies have shown that children with peanut allergy can be successfully desensitized to the offending food via OIT (7). The principle of OIT is to expose the immune system to progressively larger amounts of an allergen to induce desensitization and reduce the risks of allergic reactions after accidental ingestion of food allergen. The peanut OIT product Palforzia [Peanut (*Arachis hypogaea*) Allergen Powder-dnfp] has been approved by the FDA for the treatment of peanut allergy in children. While effective, OIT alone has some limitations. As the allergen dose increases, there is an increased risk of allergic reactions which limits the ability to escalate doses with minimal side effects. Many patients undergoing OIT continue to have dose-related side effects that can hinder their compliance and the treatment's overall efficacy (8). Furthermore, many food-allergic patients cannot tolerate OIT or fail to be desensitized, likely due to variability of cellular and molecular endotypes and clinical phenotypes (9, 10).

Omalizumab has shown promise as an adjuvant for multi-food OIT (mOIT). Patients treated with omalizumab-facilitated OIT have shown fewer adverse events (AEs) during dose escalation than those receiving OIT alone, as well as a shorter time-to-maintenance dosing (7, 11–15). However, AEs are still common during omalizumab-facilitated mOIT. Therefore, it is important to design novel therapeutic regimens based on the specific inhibition of key mediators involved in the pathogenesis of FA and anaphylaxis (16).

The Type 2 inflammatory cytokines IL-4 and IL-13 are key mediators of allergic responses and play a significant role in the pathophysiology of FA (17, 18). Dose escalations during OIT induces the up-regulation of these cytokines which in turn drives B cell isotype class switching to IgE, a phenomenon that potentially contributes to many of the dose-limiting AEs present during OIT. Dupilumab, a fully human monoclonal antibody directed against the interleukin-4 receptor alpha, blocks the activity of IL-4 and IL-13 and has demonstrated clinical efficacy in many allergic disorders such as atopic dermatitis, uncontrolled asthma, chronic rhinosinusitis with nasal polyps, and eosinophilic esophagitis. Dupilumab therapy has been shown to gradually reduce IgE levels by about 70% from baseline after 52 weeks (19). Although a recent clinical trial suggested that dupilumab alone was insufficient for the treatment of food allergy (20), use of dupilumab as an adjuvant improved the safety and efficacy of OIT (21).

To date, no study has explored the potential of combining biologics targeting different allergic mechanisms, together with mOIT, for treatment of food allergies. We designed a trial to address the efficacy and safety of the sequential use of omalizumab followed by dupilumab in multi-food allergic patients undergoing mOIT. We hypothesize that mOIT treatment with dupilumab following omalizumab will increase the likelihood of sustained unresponsiveness compared to mOIT treatment with omalizumab alone, due to the potential of dupilumab to inhibit the production of IgE in participants whose IgE would have already been substantially inhibited by pretreatment with omalizumab. Combining omalizumab with dupilumab as adjuncts to mOIT may also reduce the frequency and severity of mOIT-related AEs. Herein, we detail the clinical trial design for evaluating the safety and efficacy of omalizumab and/or dupilumab therapy in multi-food allergic patients undergoing mOIT: The Phase 2 Randomized Controlled Trial using Biologics to Improve Multi OIT Outcomes (COMBINE) study (NCT03679676).

## Methods

### Study design

**Figure 1:**
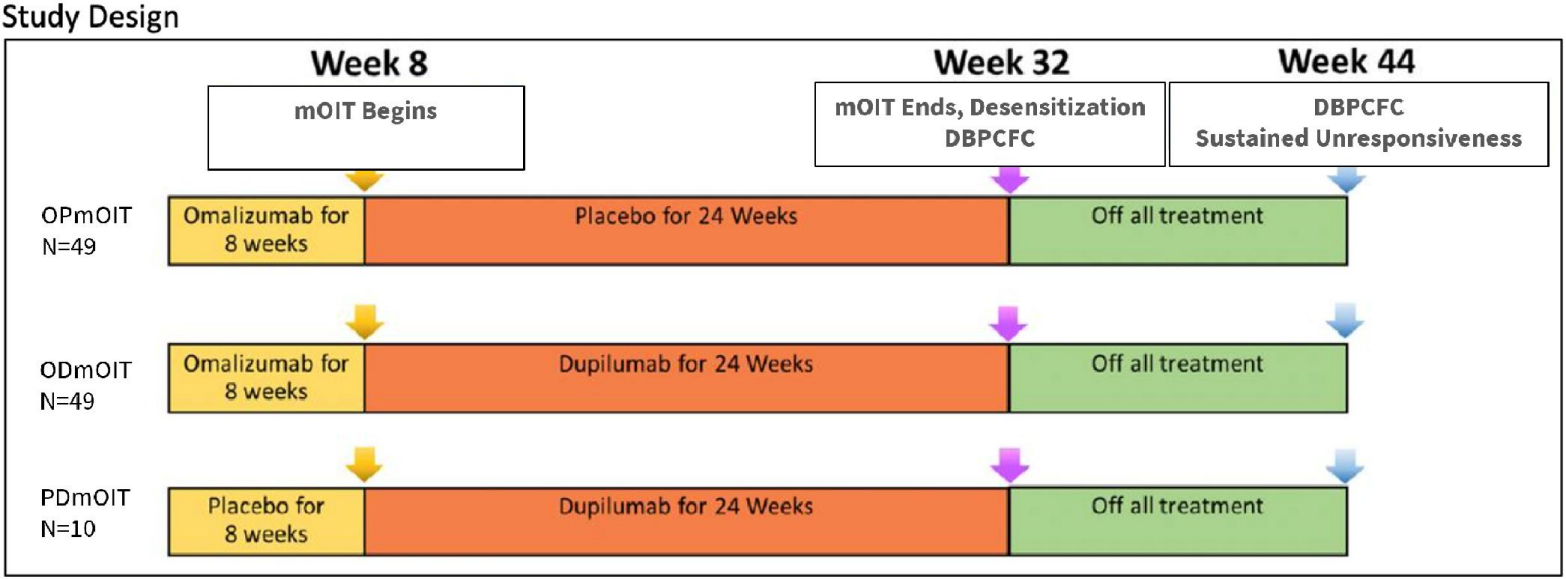
Study design of a prospective phase 2 study to test the efficacy of omalizumab and/or dupilumab in combination with mOIT.

The COMBINE Study is a 44-week, phase 2, multi-center, double-blinded, randomized, placebo-controlled trial of omalizumab and/or dupilumab with mOIT for the treatment of IgE-mediated multi-FA in participants aged 4–55 years. The study consists of a screening phase, followed by 44 weeks of active study participation with four distinct phases: run-in blockade of IgE with omalizumab, OIT dose escalation with/without blockade of IL-4/IL-13 signaling with dupilumab, maintenance dosing, and withdrawal (Figure 1). The study population is 110 participants with DBPCFC-confirmed allergies to peanut and to 2 or 3 additional protocol-specified foods, which were chosen for their relatively high prevalence (12, 15): almond, cashew, egg, fish, hazelnut, milk, sesame seed, shellfish, soy, walnut, and wheat. Inclusion and exclusion criteria are detailed in Table 1, and study-stopping rules are described in Supplementary Table S2.

**Table 1:** Inclusion and exclusion criteria.

Inclusion criteria	Individuals who meet all of the following criteria were eligible for enrollment as study participants: • Age 4 through 55 years (inclusive). • Clinical history of peanut allergy and 1 or 2 additional foods from the following foods: almond, shellfish, fish, soy, milk, cashew, hazelnut, egg, walnut, sesame seeds, and wheat. Allergy to milk and egg is defined as unable to tolerate both cooked and uncooked forms. • Positive allergy test determined by: ○ ImmunoCAP serum IgE level >4 kUA/L for each allergen within the past 12 months, or; ○ Skin prick test (SPT) ≥ 6 mm wheal diameter to each allergen. • A clinical reaction during a DBPCFC to small doses of food defined as a cumulative dose of =/<444 mg food protein. • No clinical reaction observed during the placebo (oat) challenge. • Subject and/or parent guardian must have been able to understand and provide informed consent. • Written informed consent from adult participants. • Written informed consent from parent/guardian for minor participants. • Written assent from minor participants as appropriate (e.g., at and above the age of 7 years). • For women of childbearing potential, must have agreed to remain abstinent (refrain from heterosexual intercourse) or use acceptable contraceptive methods (barrier methods or oral, injected, or implanted hormonal methods of contraception or other forms of hormonal contraception that have comparable efficacy) during the treatment period and for 60 days after the last dose of study drug.
Exclusion criteria	Individuals who meet any of these criteria were not eligible for enrollment as study participants: • History of cardiovascular disease, including uncontrolled or inadequately controlled hypertension. • Individuals less than 15 kg in weight at start of the study. • History of severe anaphylaxis to participant-specific foods that were to be used in this study, defined as neurological compromise or requiring intubation. • History of chronic disease (other than asthma, atopic dermatitis, or allergic rhinitis) that is, or is at significant risk of becoming, unstable or requiring a change in chronic therapeutic regimen. • History of eosinophilic esophagitis (EoE), other eosinophilic gastrointestinal disease, chronic, recurrent, or severe gastroesophageal reflux disease (GERD), symptoms of dysphagia (e.g., difficulty swallowing, food “getting stuck”), or recurrent gastrointestinal symptoms of undiagnosed etiology. • Severe asthma (NAEPP EPR-3 Medication Criteria Steps 5 or 6, appendix 1). • Mild or moderate asthma (NAEPP EPR-3 Medication Criteria Steps 1–4, appendix 1), if uncontrolled or difficult to control. • Uncontrolled asthma as evidenced by: ○ FEV1 < 80% of predicted, or ratio of FEV1 to forced vital capacity (FEV1/FVC) <75% of predicted, with or without controller medications (only for age 6 or greater and able to do spirometry reliably. If unable to do spirometry, PEF of >80% is acceptable), or; ○ One overnight admission to a hospital in the past year for asthma, or; ○ Emergency room (ER) visit for asthma within six months prior to screening. • Inability to tolerate biological (antibody) therapies. • Use of immunomodulator therapy (not including corticosteroids). • Use of beta-blockers (oral), angiotensin-converting enzyme (ACE) inhibitors, angiotensin-receptor blockers (ARB) or calcium channel blockers. • Unable to be adequately dosed with omalizumab based on the tables used for this study. • Current participation or within the last 4 months in any other interventional study. • Pregnancy or lactation. • Allergy to oat (placebo in DBPCFC). • Use of investigational drugs within 16 weeks of participation. • In build-up phase of immunotherapy for aeroallergens or venom. • Past or current medical problems or findings from physical examination or laboratory testing that are not listed above, which, in the opinion of the investigator, may have posed additional risks from participation in the study, may interfere with the participant's ability to comply with study requirements, or that may impact the quality or interpretation of the data obtained from the study. • Known hypersensitivity to omalizumab or any of its excipients. • Known hypersensitivity to dupilumab or any of its excipients.

Participants will be recruited across three study centers in California, United States: Stanford University, Palo Alto; the University of California Los Angeles, Los Angeles; and the University of California San Diego, La Jolla. Study objectives and endpoints are fully detailed in Table 2, with planned mechanistic assays described in Table 3 (additional details in the Supplementary Methods). A full schedule of study events, visits, and procedures is provided in detail in Table 4.

**Table 2:** Schedule of events.

	Screen	Omalizumab or placebo 8 weeks	Initial Dose escalation day (IDED)	Day after initial dose escalation day (IDED + 1)	Buildup phase w/ dupilumab or placebo 24 weeks	Maintenance phase	Withdrawal phase 12 weeks
*Time*	*Day −270–0*	*Every 2–4 wks (Week 0–8)*	*Week 8*	*1 day after IDED (week 8, day 1)* ^d^	*Every 2 wk (Weeks 10–32)*	*Week 32*	Week 44 *End of Study*
*Visit window*	± *12 weeks*	± *7 days*	*± 7 days*	*+ 6 days*	*± 7 days*	*± 14 days*	*± 14 days*
Informed consent	X						
Medical history	X						
Physical assessment	X	X^c^	X	X	X	X	X
Con meds	X	X	X	X	X	X	X
Adverse events	X	X	X	X	X	X	X
Specific IgE/IgG4	X						X
Skin prick testing	X					X	X
CBC with differential	X		X			X	X
Blood for mechanistic studies	X	X	X			X	X
Serum pregnancy test	X						
Lung function	X		X	X	X	X	X
Diaries		X	X	X	X	X	X
Inject epi training	X						X
Omalizumab or dupilumab injection^a^		X	X		X		
Optional samples^b^	X		X			X	X
DBPCFC	X					X test desensitization	X test SU
OIT			X	X	X	X	
Questionnaires	X		X			X	X

^a^ Omalizumab or placebo will be given every 2 or 4 weeks from week 0 until week 8. Dupilumab or placebo will be given every 2 to 4 weeks from week 10 to week 32.

^b^ Optional Samples include: buccal swabs, skin swabs, saliva samples, fecal samples, urine samples.

^c^ Symptom-directed physical assessment.

^d^ IDED + 1 procedures to be done if dosing is performed in clinic (as applicable).

**Table 3:** Summary of study objectives and associated endpoints.

Level	Objective	Endpoint
Primary	To determine whether the suppression of allergic responses by an anti-IgE antibody (omalizumab) followed by the combination of multi-OIT (mOIT) with concomitant IL-4R*α* blockade (dupilumab) can increase the ability to sustain clinical tolerance in the absence of continued therapy	Three co-primary endpoints via hierarchical design, with each compared between Cohort A and Cohort B. These include success* rates of passing a DBPCFC at Week 44 to the following: 1. Peanut 2. Peanut and at least one other food allergen 3. Peanut and two other food allergens *Success/passing defined as the consumption of ≥1,043 mg cumulative protein to a food with no or mild objective reactions only
Secondary	Clinical: To compare the ability of the different treatment regimens (Cohort A: omalizumab/placebo for dupilumab + mOIT; Cohort B: omalizumab/dupilumab + mOIT; and Cohort C: placebo for omalizumab/dupilumab + mOIT) to desensitize participants to different thresholds and quantities of foods	1. Proportion of participants in each Cohort achieving a 10-fold change in the cumulative tolerated dose of each allergen at Week 32 and/or 44 DBPCFC, compared to baseline 2. Proportion of participants in each Cohort who pass DBPCFCs for each allergen at a cumulative dose of ≥1,043 mg, ≥ 2,043 mg, or ≥4,043 mg protein at Week 32 and/or 44 DBPCFCs
	Clinical: To compare treatment success between different food allergens at Week 32	Proportion of participants in each Cohort who successfully pass DBPCFCs to a cumulative dose of ≥2,043 mg protein to 1, 2, or 3 allergens, when applicable, at Week 32 DBPCFCs
	Clinical: To compare the ability of the different treatment regimens to induce sustained unresponsiveness (SU) to one or more foods	Proportion of participants in each Cohort who successfully pass DBPCFCs to a cumulative dose of ≥1,043 mg protein to 1, 2, or 3 allergens, when applicable, at Week 44 DBPCFCs (SU)
	Safety: To assess treatment safety during both active treatment and following withdrawal	1. Frequency of AEs, SAEs, and safety events in each cohort during the first 32 weeks of treatment 2. Frequency of AEs, SAEs, and safety events among treatment cohorts after completing their mOIT withdrawal to Week 44 or end of study participation
Mechanistic/exploratory	To evaluate the immunological responses associated with success and failure with respect to treatment and SU outcomes	Differences in immunological responses, as measured by allergen-specific and non-specific markers, such as free allergen-specific IgE, total IgE, specific IgG4, specific IgG4/IgE ratios, basophil activation tests, basophil phenotyping, B cell receptor repertoire features, B cell phenotyping, T cell receptor levels, T cell phenotyping, and other immune-related cells measured at: 1. Baseline 2. IDED (Week 8) 3. End of maintenance phase (Week 32) 4. End of withdrawal phase (Week 44)
Exploratory	To evaluate quality of life markers during both active treatment and following withdrawal	1. Quality of life questionnaires at baseline, Week 32, and Week 44 2. Time to maintenance multi-OIT dose by Cohort and by number of allergens included in the multi-OIT

**Table 4:** Summary of planned mechanistic assays.

Assay	Panel/parameter
Serum	Specific IgA
	Specific & total IgE
	Specific IgG4
Cell components (cytometry by time of flight)	T helper 1 cells
	T helper 2 cells
	T helper 17 cells
	Natural killer T cells
	T regulatory cells
	Dendritic cells (TSLP receptor, CD103, CCR9)
	Cell death markers
	Chemokine receptors (CCR4, CCR8)
	Allergen-specific cells (Th1, Th2, Treg)
Basophil activation	Basophil reactivity (CD203c/CD63)
B cell receptor repertoire	B cell clones expressing IgE and IgG4

### Randomization and omalizumab/placebo treatment (weeks 0–8)

Participants meeting all eligibility criteria will proceed to Week 0 and undergo randomization in a 5:5:1 ratio to one of three cohorts using block randomization, stratified by number of allergens included in their mOIT treatment. The COMBINE trial is fundamentally a two-arm superiority study designed to test whether adding dupilumab to omalizumab-facilitated mOIT improves outcomes relative to omalizumab-facilitated mOIT alone. All primary and secondary analyses compare Cohorts A and B only:

1. Cohort A (*n* = 50) will receive omalizumab for 8 weeks followed by 24 weeks of treatment with mOIT and placebo for dupilumab, then 12 weeks with no treatment.
2. Cohort B (*n* = 50) will receive omalizumab for 8 weeks followed by treatment with mOIT and dupilumab for 24 weeks, then 12 weeks with no treatment.

A third, mechanistic-only arm, Cohort C, is included and serves exclusively as a mechanistic comparator to explore dupilumab's effects in the absence of prior omalizumab treatment. Cohort C (*n* = 10) will receive placebo for omalizumab for 8 weeks, followed by treatment with mOIT and dupilumab for 24 weeks, then 12 weeks with no treatment. Given the small sample size, this Cohort lacks statistical power for reliable efficacy and safety analysis and will not be included in such.

Beginning at Week 0, participants will be dosed with omalizumab or placebo for omalizumab every 2 or 4 weeks according to their Week 0 body weight and most recent total IgE value. The weight- and IgE-based dosing algorithm targets 0.008 or 0.016 mg omalizumab/kg/(kUA/L) every 2 or 4 weeks, respectively, while not exceeding 20 mg/kg in a single administration, similar to the FDA-approved dosing tables for asthma (Supplementary Table S1). All doses of omalizumab/omalizumab placebo (as well as dupilumab/dupilumab placebo) will be dispensed to and administered by an unblinded injector in a fashion that maintains treatment blinding (i.e., shielded from the view of participants, visitors, and all other blinded individuals). All participants will be observed for 2 h after the first three omalizumab or placebo injections they receive in clinic and 30 min for injections thereafter from Week 0–8.

### Initial dose escalation day (week 8)

At Week 8, each participant will receive their final dose of omalizumab/placebo and, following observation, undergo an Initial Dose Escalation Day (IDED) for all foods to be included in their mOIT, starting at a dose of 1 mg protein per food (combined in a single mix) and escalating to 10 mg, 50 mg, 100 mg, 210 mg, 420 mg, and 1,000 mg protein per food sequentially as tolerated (Table 5). This IDED is designed to determine each participant's current tolerance and level in which they may begin mOIT dosing. If a participant does not tolerate at least 1 mg protein of each of their foods at IDED, they shall be deemed a treatment failure and will not receive further treatment nor undergo subsequent food challenges.

**Table 5:** IDED and OIT dose escalation schedule.

Dose sequence	Protein dose (mg)		
	Per allergen	Two-allergen multi-OIT^a^	Three-allergen Multi-OIT^a^
1	1	2	3
2	10	20	30
3	50	100	150
4	100	200	300
5	210	420	630
6	420	840	1,260
7	1,000	2,000	3,000

^a^ Total protein dose divided equally among included allergens.

### mOIT and dupilumab/placebo treatment (weeks 8–32)

Following the Week 8 IDED, participants will begin daily mOIT dosing. Each participant will receive the highest tolerated dose at IDED as their home dose and return to the clinic every two weeks to attempt an observed dose escalation up to a maximum dose of 1,000 mg protein per allergen. The dose escalation schedule will follow that of the IDED (Table 5). Participants tolerating 1,000 mg protein of each food at the IDED will maintain that dose until Week 32. Dose escalation attempts will continue until Week 30 or until the participant reaches and tolerates 1,000 mg protein per allergen, whichever occurs first. Prior to each in-clinic dose escalation, participants shall be instructed to withhold their daily home mOIT dose and any prophylactic antihistamines. Throughout mOIT dosing, the participant's dose may be decreased as needed in select cases, such as intolerance or illness, and escalation attempts may be postponed for 1 or 2 visits based on clinical judgement; however, if a dose escalation attempt is not made within three consecutive scheduled clinic visits or the participant fails to successfully tolerate a dose escalation within three consecutive attempts, dosing will be discontinued and the participant shall be deemed a treatment failure. This rule will not apply in cases wherein the delay in escalation is due to administration of epinephrine or illness. Beginning at Week 10, participants will receive concomitant treatment with dupilumab or placebo dosed every 2–4 weeks according to FDA-approved dosing guidelines for atopic dermatitis, extended to those aged 4 years or older. The final dose of dupilumab or placebo will be administered at the Week 30 visit.

### Maintenance phase (weeks 30–32, minimum)

Upon reaching 1,000 mg protein per allergen, participants will maintain their mOIT dose with no further escalation. The maintenance phase for each participant begins when the participant reaches 1,000 mg protein per food and continues through the end of their Week 32 DBPCFCs. Each participant must escalate to and tolerate 1,000 mg protein per allergen on or before the end of their Week 30 visit; those unable to meet these criteria shall bedeemed a treatment failure and will not receive further study treatment nor undergo subsequent food challenges.

### Week 32 DBPCFCs (week 32)

At Week 32, participants will undergo DBPCFCs to each allergen in their mOIT plus placebo on separate days while continuing daily mOIT dosing between challenge days. Each DBPCFC will consist of 8 doses given every 15–30 min in increasing amounts up to a cumulative total of 4,043 mg food protein or placebo as tolerated: 3 mg, 10 mg, 30 mg, 100 mg, 300 mg, 600 mg, 1,000 mg, and 2,000 mg protein. If the participant fails to tolerate at least 1,043 mg cumulative protein of at least one food allergen during the DBPCFCs, they shall be deemed a treatment failure and will not undergo subsequent food challenges. Participants tolerating at least one active food challenge at cumulative dose of at least 1,043 mg protein will continue into the withdrawal phase.

### Withdrawal phase and week 44 DBPCFCs (week 32–44)

Following each participant's final Week 32 DBPCFC, those eligible to continue to the withdrawal phase will discontinue mOIT and dupilumab or placebo dosing through Week 44. This phase is designed to examine mechanisms underlying sustained unresponsiveness (SU). At Week 44, after 12 weeks off treatment, participants will undergo additional DBPCFCs to the specific foods to which they were desensitized (i.e., tolerated ≥1,043 mg cumulative protein) at Week 32 and placebo on separate days**.** The design and procedures for the Week 44 DBPCFCs are identical to those at Week 32. Each participant's sensitivity to food allergen will be defined as the dose at which the participant experiences dose-limiting allergic reactions as per the Consortium for Food Allergy Research Grading Scale for Systemic Allergic Reactions v.3.0 (22). Participants who pass one or more food challenges, defined as experiencing no or mild objective reactions to a cumulative dose of ≥1,043 mg protein per food allergen, at Week 44 will be considered to have achieved SU for the associated food allergen and completed the study. If the participant fails to pass the Week 44DBPCFC for all foods tested, they shall be deemed an SU failure.

## Statistical considerations

### Analysis populations

The intent-to-treat (ITT) sample will include all participants who are enrolled. The safety sample is defined as all enrolled participants who receive at least one dose of mOIT. Participants in the safety sample will be analyzed according to the treatment that they actually received and will be utilized to assess differences in safety endpoints.

### Primary analyses

The primary analysis will be an ITT comparison of SU success rates between participants who received omalizumab plus mOIT with placebo for dupilumab (Figure 1, Cohort A) and participants who received omalizumab plus mOIT with dupilumab (Cohort B), where SU success is defined as the consumption of at least 1,043 mg cumulative protein at Week 44 DBPCFC with no or mild objective reactions (Table 2). A hierarchical endpoint analysis will be used, with peanut allergy as primary, to test the difference in proportions of participants who pass the peanut DBPCFCs between Cohorts A and B at Week 44. If this test is significant, the subsequent comparison will be a test between the proportions who pass peanut and at least one other food allergen. If this second test is significant, a third comparison will be made between Cohorts A and B of the proportion who pass all three DBPCFCs to active allergens. Missing tests or dropouts will be considered treatment failures and will be imputed as allergic cases. For all analyses, a two sided Fisher's exact test with an alpha level of 0.05 will be leveraged to test for significance. Based on results from the MTAX study (15), 26% of participants in Cohort A and 62% in Cohort B are expected to tolerate 1,043 mg of peanut protein at the Week 44 SU timepoint. Given this assumption, a cohort size of at least 43 participants per arm would provide a superior power of 93% at alpha level of 0.05 using the two-sided chi-square test. Considering a 15% dropout, 50 participants per arm is expected to result in sufficient power to detect the expected difference.

Supportive analyses of the primary endpoint will also be conducted, including a multivariable logistic regression for each of the three co-primary endpoint comparisons between Cohorts A and B. The model will be adjusted for important baseline demographic and clinical characteristics, such as age and number of original FAs that participants start with. If the first test uses exact logistic regression, the subsequent test will also be exact version. Further discussion of secondary, exploratory, mechanistic, descriptive analyses and power calculations may be found in the supplemental text.

## Discussion

The COMBINE study is the first study to evaluate the combination of two biologics, omalizumab and dupilumab, as adjuvants for mOIT. Although omalizumab-facilitated mOIT is effective for the treatment of multi-FA, the treatment burden of mOIT remains high and SU is only achieved in a fraction of participants. The reduction of GI symptoms in dupilumab-facilitated mOIT (21) suggests that dupilumab may help alleviate mOIT-associated AEs that persist in the setting of omalizumab-facilitated mOIT. We believe that targeting IgE both directly with omalizumab and indirectly with dupilumab will enhance the safety and long-term efficacy of mOIT. If correct, dupilumab may be leveraged to support patients who would otherwise struggle with omalizumab-facilitated mOIT.

The COMBINE study also aims to explore several mechanistic questions related to mOIT, including whether dupilumab is able to enhance immunomodulatory effects of mOIT by decreasing Type 2 responses and increasing the food allergen-specific IgG response. These effects carry the potential to improve the safety and tolerability of mOIT, particularly during dose escalation, as well as improve the efficacy of mOIT in promoting SU. In addition, the study will evaluate whether dupilumab influences biomarkers and processes known to be critical to the allergic response, including its ability to reduce allergen-specific immunoglobulin sub-class switching to IgE, basophil activation, and Th2 cytokine levels.

There are several limitations associated with this study. First, while we include a third arm, Cohort C, set to receive dupilumab without prior omalizumab for mechanistic exploration, this arm is notably underpowered for formal comparative analyses of safety and efficacy and, thus, will not be included in such. Furthermore, this study was designed before the conclusion of two other clinical trials evaluating the use of dupilumab in food allergy suggesting that dupilumab monotherapy is insufficient for the treatment of food allergy (20) but does have potential for use as an adjuvant for mOIT (21). In light of data from these publications, COMBINE may have been designed with a shorter duration of dupilumab treatment than would be required to achieve the primary endpoint. Future studies investigating the potential of longer treatment with dupilumab would be valuable. Finally, this study is also limited by the requirement for peanut as an allergen in all participants mOIT. This requirement is included in an effort to ensure that there will be sufficient participants to power planned analyses for at least one allergen; however, many patients with multiple food allergies may not be allergic to peanut, and further research may be needed to investigate the safety and efficacy in such patients.
