# Supplementary material for: A phase 2 randomized controlled trial using biologics to improve multi OIT outcomes (COMBINE): design, rationale, and methods
Source: Front Allergy. 2025 Dec 18;6:1729111. doi: 10.3389/falgy.2025.1729111 (PMC12756116; doi:10.3389/falgy.2025.1729111)
Supplement: Supplementary file 1 [file Datasheet1.docx]

**Supplemental Text:**

**Supplemental Methods**

*Multi-allergen oral immunotherapy*

Multi-allergen OIT dosing kits are manufactured by the Manufacturing Facility at the Sean N. Parker Center for Allergy & Asthma Research with Stanford University (Mountain View, CA). Each dose of oral immunotherapy is supplied as a dry powder consisting of the participant’s specific two- or three-allergen mix weighed out and packaged into individual unit doses based on the total protein content of each of the parent allergen flours/powders and the target total protein dose of the final unit.

*Skin prick testing & biosample collection*

Skin prick testing will be conducted at screening, Week 32, and Week 44. Blood samples will be collected at screening and Weeks 8, 32, and 44 for complete blood count with differential, and at screening and Weeks 0, 8, 32, and 44 for mechanistic studies. For tests and samples taken at Weeks 32 and 44, each test and sample will be conducted or collected prior to the participant’s first DBPCFC.

*Physical examination & lung function test*

Prior to any DBPCFC, IDED, or OIT dose escalation attempts, participants will undergo physical assessment by a trained clinician of the study team that includes lung function evaluation via spirometry or peak flow. Dosing will not proceed if the participant displays active wheezing, diminished lung function testing, or a current flare of atopic dermatitis that contraindicates dosing in the clinical judgment of the study physician.

*Oral food challenge*

All food challenges will be performed under physician supervision. The participant and all research staff are blinded to the identity of the challenge material. For each DBPCFC, the placebo (oat) is dosed at volumes approximately equal to that of the corresponding peanut dose. Doses will be given every 15-30 minutes in increasing amounts according to the DBPCFC dosing schedule. If the study team suspects a reaction may be developing, they may exercise their clinical judgment to separate doses by up to an additional 30 minutes. Reactions will be scored according to the Consortium for Food Allergy Research (CoFAR) Grading Scale for Systemic Allergic Reactions v.3.0 (**Supplemental Table** **S3**). If the participant is observed to have any symptoms deemed to be dose-limiting, the food challenge will be terminated, and the subject will be given appropriate treatment (**Supplemental Table S4**). Each participant will be observed for a minimum of two hours after the final administered dose and discharged only when deemed clinically stable by a study clinician.

*Quality of Life Questionnaires (QoL)*

*Participant QoL questionnaires are collected at baseline, week 32 and week 44. These questionnaires will be compared between time points using the Wilcoxon signed-rank test and compared between the three cohorts by using the Whitney U test.*

***Screening (Weeks -24 to 0)***

Informed consent and assent, if applicable) are obtained before proceeding with any trial procedures. During the screening phase, each participant will be screened according to the established eligibility criteria (**Table 1**). Participants will initially be screened against all eligibility criteria excluding the DBPCFCs. If participants meet all criteria they will undergo DBPCFCs to each of their potential allergens, including peanut and two or three other protocol-specified allergens, as well as placebo, each performed on separate days. Each screening DBPCFC will consist of 6 doses given in increasing amounts up to a cumulative total of 444 mg of food allergen protein or equivalent volume of placebo (oat) as tolerated: 1 mg, 3 mg, 10 mg, 30 mg, 100 mg, and 300 mg protein. Participants must have a positive reaction at or before the 300 mg protein (444 mg cumulative protein) dosing level of peanut and at least one other allergen to remain eligible for the study and proceed to randomization.

**Statistical and Sample Size Considerations - Continued***Please see the main manuscript text for statistical and sample size considerations specific to the primary analyses.*

***Secondary analyses***

The secondary endpoints for Cohorts A vsB, will be compared within the ITT population. All secondary efficacy endpoints will be analyzed using two-sided Fisher's exact test (or Pearson chi-square test if all expected cell counts are ≥5) with α=0.05. Success rates in each arm will be estimated with exact binomial 95% confidence intervals. Odds ratios comparing Cohort B to Cohort A will be calculated with exact 95% confidence intervals.

Multivariable logistic regression models will be conducted and adjusted for age and number of original food allergens that participants start with. The ordinal comparisons for number of allergies will also be adjusted by age and number of allergens in each participant’s multi-OIT. The number of allergies will be modeled under the proportional odds assumption; however, this assumption will be tested, and a partial proportional odds model will be used if this assumption is violated.

We will additionally calculate the proportion of participants who have a 10-fold change in the cumulative tolerated dose for each allergen at Weeks 32 and/or Week 44 compared to baseline. The difference of the proportions across allergens will be compared within each Cohort. The proportions for each food will also be compared across treatment arms. The analysis will be conducted using the chi-square test or Fisher’s exact test.

Proportions of AEs and accidental food allergen exposure will additionally be tabulated using the safety sample for each cohort.

***Mechanistic & Exploratory Analyses***

Additional tertiary mechanistic endpoints will be evaluated using immunological assays. The exploratory mechanistic endpoints will be compared among Cohorts A, B, and C (**Table 2**). There are numerous questions of interest that involve the utility of data generated on a variety of platforms for predicting clinical response or for describing other relevant clinical phenotypes that could provide insight into the mechanisms of OIT. An important goal is to identify those with potential ability to discriminate across phenotypes relevant for characterizing and treating patients with significant FA. Thus, for each platform of interest, we will evaluate the roles of mechanistic features in predicting Week 32 and Week 44 success rates and control the false discovery rate to be no more than 5%. In addition to statistical testing, analyses (e.g., those that employ Least Absolute Selective Shrinkage Operator) will jointly evaluate features and identify those with relatively more importance with respect to the clinical phenotype of interest. Such methods are appropriate when jointly considering a large number of correlated features, as we anticipate with data generated from the CyTOF platform. Additionally, other analyses such as hierarchical clustering will provide graphical depictions of clustering of features and of subjects, potentially providing insight into features within and across platforms and potentially meaningful clinical phenotypes. We will also evaluate the associations between clinical and lab characteristics and clinical outcomes. Participant quality of life questionnaires will be compared between time points and between three cohorts. The quality of life scores obtained from the questionnaires will be compared between Cohorts using the Mann-Whitney U test and between time points using the Wilcoxon signed-rank test. The time-to-maintenance OIT dose will be compared by Cohort and number of allergens in the participant’s multi-OIT via the log-rank test and Kaplan-Meier plot.

***Descriptive Analyses***

Descriptive statistics will be presented and compared among Cohorts A, B, and C. Means, medians, standard deviations, and interquartile ranges will be presented for continuous variables. Frequency tables will be provided for categorical and discrete terms, such as family history and ethnicity. Graphical tools such as boxplots and histograms will be used to assess distributional properties of continuous variables.

***Power and Sample Size Calculations***

The primary endpoint is a comparison of SU rates at Week 44 for omalizumab and placebo for dupilumab plus OIT (Cohort A) compared to omalizumab and dupilumab plus OIT (Cohort B). To carry out power calculations for the primary endpoint, we used prior data from our published MTAX study^1^. This study investigated multi-OIT facilitated by 16 weeks of omalizumab. Evaluation of SU occurred at Week 36 DBPCFCs following successful desensitization at Week 30 and 6 weeks of decreased maintenance dose or avoidance of multi-OIT. At Week 36, a significantly greater proportion of the participants on active OIT (34 of 40; 85%) passed a DBPCFC, defined as tolerating at least 2000 mg cumulative protein, for two or more of their offending foods compared to those avoiding OIT (11 of 20, 55%; odds ratio 4.5, 95% CI 1.1–19.3, p=0.03). Further, the MTAX Study suggested rates of success were approximately equal across allergens Weeks 30 and 36.Therefore, we assumed that the foods would behave approximately similarly in the trial described herein.

In this study, we apply a fixed-sequence procedure for three co-primary endpoints to be tested hierarchically. Power and sample size calculations were conducted for each of the three tests based on simulation in R (version 3.6.0). The MTAX study^1^ previously reported pairwise correlations between 0.64 to 0.84 for Week 36 outcomes, including passing DBPCFCs for peanut alone, for peanut plus at least one other FA, and for peanut plus at least two other FAs. As such, we assume that the correlation between three endpoints in this study is roughly 0.75. Based on the proportions of participants in the MTAX study who passed peanut and other FAs at the SU visit (after 6 weeks of avoidance), we expect that there will be 26% in Cohort A and 62% in Cohort B who successfully pass peanut at Week 44 SU testing in the COMBINE study 12 weeks after discontinuing treatment. Given these effect sizes, we estimate that at least 43 participants per arm will provide a superior power of 93% at alpha level of 0.05 using the two-sided chi-square test for the primary comparison between Cohorts A and B,. Assuming that 25% and 59% for Cohorts A and B, respectively, pass peanut and at least one other FA in the subsequent test, 43 participants per arm will be sufficient to achieve 86% power. If subsequently fewer participants were to pass all three FAs, for example 24% and 58% for Cohorts A and B, respectively, power would be reduced to 81%. Considering a 15% dropout rate, 50 participants per arm is expected to result in sufficient power to detect the above differences. Using Fisher’s exact test, the power would be 88%, 79%, and 73% to detect the estimated effect sizes stated above, respectively, if all tests are performed. The power calculation for the third test is based on the scenario in which all participants receive mOIT for three foods.

**Supplemental Tables**

**Table S1.** Omalizumab dosing schedule - Omalizumab doses (milligrams/dose) administered by subcutaneous injection every 2 weeks (A) or 4 weeks (B)

**A)**

| Screening IgE (IU/ml) | Body weight (kg) | | | | | | | | | | |
| --- | --- | --- | --- | --- | --- | --- | --- | --- | --- | --- | --- |
|  | 15-20 | 21-25 | 26-30 | 31-40 | 41-50 | 51-60 | 61-70 | 71-80 | 81-90 | 91-125 | 126-150 |
| 101-200 |  |  |  |  |  |  |  |  |  | 225 mg | 300 mg |
| 201-300 |  |  |  |  |  |  | 225 mg | 225 mg | 225 mg | 300 mg | 375 mg |
| 301-400 |  |  |  |  | 225 mg | 225 mg | 225 mg | 300 mg | 300 mg | 450 mg | 525 mg |
| 401-500 |  |  |  | 225 mg | 225 mg | 300 mg | 300 mg | 375 mg | 375 mg | 525 mg | 600 mg |
| 501-600 |  |  |  | 225 mg | 300 mg | 300 mg | 375 mg | 450 mg | 525 mg | 600 mg |  |
| 601-700 |  |  | 225 mg | 225 mg | 300 mg | 375 mg | 450 mg | 525 mg | 600 mg |  |  |
| 701-800 | 225 mg | 225 mg | 225 mg | 300 mg | 375 mg | 450 mg | 450 mg | 600 mg |  |  |  |
| 801-900 | 225 mg | 225 mg | 225 mg | 300 mg | 375 mg | 450 mg | 525 mg |  |  |  |  |
| 901-1000 | 225 mg | 225 mg | 300 mg | 375 mg | 450 mg | 525 mg | 600 mg |  |  |  |  |
| 1001-1100 | 225 mg | 225 mg | 300 mg | 375 mg | 450 mg | 600 mg |  |  |  |  |  |
| 1101-1200 | 300 mg | 300 mg | 300 mg | 450 mg | 525 mg | 600 mg |  |  |  |  |  |
| 1201-1300 | 300 mg | 300 mg | 375 mg | 450 mg | 525 mg |  |  |  |  |  |  |
| 1301-1500 | 300 mg | 300 mg | 375 mg | 525 mg | 600 mg |  |  |  |  |  |  |
| 1501-2000 | 375 mg | 450 mg | 600 mg | 600 mg | 600 mg |  |  |  |  |  |  |

**B)**

| Screening IgE (IU/ml) | Body weight (kg) | | | | | | | | | | |
| --- | --- | --- | --- | --- | --- | --- | --- | --- | --- | --- | --- |
|  | 15-20 | 21-25 | 26-30 | 31-40 | 41-50 | 51-60 | 61-70 | 71-80 | 81-90 | 91-125 | 126-150 |
| 30-100 | 75 mg | 75 mg | 75 mg | 75 mg | 150 mg | 150 mg | 150 mg | 150 mg | 150 mg | 300 mg | 300 mg |
| 101-200 | 150 mg | 150 mg | 150 mg | 150 mg | 300 mg | 300 mg | 300 mg | 300 mg | 300 mg |  |  |
| 201-300 | 150 mg | 150 mg | 150 mg | 225 mg | 300 mg | 300 mg |  |  |  |  |  |
| 301-400 | 225 mg | 225 mg | 225 mg | 300 mg |  |  |  |  |  |  |  |
| 401-500 | 225 mg | 225 mg | 300 mg |  |  |  |  |  |  |  |  |
| 501-600 | 300 mg | 300 mg | 300 mg |  |  |  |  |  |  |  |  |
| 601-700 | 300 mg | 300 mg |  |  |  |  |  |  |  |  |  |

**Table S2.** Study stopping rules.

| ***Individual stopping rules*** |
| --- |
| Participants may be prematurely terminated from the study for any of the following reasons: |
| 1.      The participant elects to withdraw consent from all future study activities, including follow-up |
| 2.      The participant is “lost to follow-up” (i.e., no further follow-up is possible because attempts to reestablish contact with the participant have failed) |
| 3.      The participant dies |
| 4.      The Investigator no longer believes participation is in the best interest of the participant |
| 5.      Individual safety stopping rules:   1. Anaphylaxis resulting in hypotension, neurological compromise or mechanical ventilation secondary to OIT dosing, injections, or any food challenge 2. The subject develops biopsy-documented eosinophilic esophagitis with synchronous symptoms or other eosinophilic gastrointestinal disease 3. Any subject deemed to have severe allergic reactions and who receives aggressive therapy (e.g., mechanical ventilation, three or more doses of epinephrine for a life-threatening reaction) at any time should be discontinued from further therapy 4. Other circumstances including, but not limited to, the following:    - 1. Poor control or persistent activation of secondary atopic disease (e.g., asthma or atopic dermatitis)      2. Started on beta-blockers, or other prohibited medications, with no alternative medications available per the prescribing physician      3. Pregnancy |
| ***Study stopping rules*** |
| The study may be prematurely terminated for any the following reasons: |
| 1.      If the Investigator, Medical Monitor, or the National Institute of Allergy and Infectious Diseases (NIAID) Medical Officer discover conditions that indicate that the study should be discontinued, an appropriate procedure for stopping the study pending NIAID Data and Safety Monitoring Board review will be instituted, including notification of the FDA and Institutional Review Board. |
| 2.      If any of the stopping rules listed below are met, study enrollment and initial dose escalation days will be suspended, oral immunotherapy dose escalation will be paused, and all enrolled participants will remain on their current OIT dose pending expedited review of all pertinent data:   1. Any death related to dosing or study procedure 2. More than three participants requiring more than two injections of epinephrine during a single oral immunotherapy dosing 3. More than 1 case of CoFAR Grade 4 AE (**Supplemental Table S3**) related to food allergen dosing 4. More than 2 cases of CoFAR Grade 4 AE (**Supplemental Table S3**) related to an oral food challenge 5. More than 3 serious adverse events (SAEs) related to investigational product 6. More than 3 cases of eosinophilic esophagitis with synchronous clinical symptoms and confirmatory biopsy findings |
| 3.      If more than one participant requires more than two injections of epinephrine during a single omalizumab/placebo or dupilumab/placebo injection, injections will be paused and participants will remain on their current oral immunotherapy dose pending expedited review of all pertinent data |

**Table S3.** The Consortium for Food Allergy Research Grading Scale for Systemic Allergic Reactions v.3.0

| **Grade 1** | **Grade 2** | **Grade 3** | **Grade 4** | **Grade 5** |
| --- | --- | --- | --- | --- |
| Reaction involving **one of the following organ systems in which the symptoms are mild**:  Cutaneous Generalized pruritus, generalized urticaria, flushing, angioedema  Upper respiratory Rhinitis, cough unrelated to laryngeal edema or bronchospasm  Conjunctival Injection/redness, itching, tearing  GI  Nausea, abdominal pain (no change in activity level), single episode of vomiting and/or single episode of diarrhea | Reaction involving **two or more of the following organ systems in which the symptoms are mild**:  Cutaneous  Generalized pruritus, generalized urticaria, flushing, angioedema  Upper respiratory Rhinitis, cough unrelated to laryngeal edema or bronchospasm  Conjunctival Injection/redness, itching, tearing  GI  Nausea, abdominal pain (no change in activity level), single episode of vomiting, and/or single episode of diarrhea  **OR**  Reaction involving **at least one of the following organ systems in which the symptoms are moderate:**  Cutaneous  Generalized pruritus, generalized urticaria, flushing, angioedema  Upper respiratory Rhinitis, cough unrelated to laryngeal edema or bronchospasm  Conjunctival Injection/redness, itching, tearing  GI  Nausea, abdominal pain (with change in activity level), two episodes of vomiting and/or diarrhea | Reaction involving **one or more of the following organ systems**:  Lower respiratory Throat tightness, wheezing, chest tightness, dyspnea, cough that responds to short-acting bronchodilator treatment (including IM epinephrine) with or without supplemental oxygen  GI  Severe abdominal pain, more than two episodes of vomiting and/or diarrhea | **Life-threatening reaction** involving **one or more of the following organ systems** with or without other symptoms listed in Grades 1 to 3:  Lower respiratory  Throat tightness with stridor, wheezing, chest tightness, dyspnea, or cough associated with a requirement for supplemental oxygen and refractoriness to short-acting bronchodilator treatment (including IM epinephrine)^1^  **OR**  Respiratory compromise requiring mechanical support  Cardiovascular Reduced blood pressure with associated symptoms of end-organ dysfunction (e.g., hypotonia [collapse], syncope) defined as:   - Children: low systolic BP (age specific)2 or >30% decrease in systolic BP - Adults: systolic BP of less than 90 mmHg or >30% decrease from baseline | Death |

**Table S4.** Definitions of dose-limiting symptoms

| **Mild** | **Moderate** | **Severe** |
| --- | --- | --- |
| Cutaneous  Limited (few) or localized hives, swelling (e.g., mild lip edema), skin flushing (e.g., few areas of faint erythema) or mild pruritus (e.g., occasional scratching)  Respiratory  Rhinorrhea (e.g., occasional sniffling or sneezing), nasal congestion, occasional cough, throat discomfort  GI  Mild abdominal discomfort (including mild nausea with or without decreased activity), isolated emesis thought to be secondary to gag | Cutaneous  Systemic hives (e.g., numerous or widespread hives), swelling (e.g., significant lip or face edema), pruritus causing protracted scratching, more than a few areas of erythema or pronounced erythema  Respiratory  Throat tightness without hoarseness, persistent cough, wheezing without dyspnea  GI  Persistent moderate abdominal pain/cramping/nausea with decreased activity, vomiting | Cutaneous  Severe generalized urticaria/angioedema/erythema  Respiratory  Laryngeal edema, throat tightness with hoarseness, wheezing with dyspnea, stridor  GI  Severe abdominal pain/cramping/repetitive vomiting  Neurological  Change in mental status  Circulatory  Clinically significant hypotension |

1. Andorf S, Purington N, Kumar D, et al. A Phase 2 Randomized Controlled Multisite Study Using Omalizumab-facilitated Rapid Desensitization to Test Continued vs Discontinued Dosing in Multifood Allergic Individuals. *EClinicalMedicine.* 2019;7:27-38.
